# Chlorogenic Acid Protects against Advanced Alcoholic Steatohepatitis in Rats via Modulation of Redox Homeostasis, Inflammation, and Lipogenesis

**DOI:** 10.3390/nu13114155

**Published:** 2021-11-20

**Authors:** Vyacheslav Buko, Ilya Zavodnik, Grażyna Budryn, Małgorzata Zakłos-Szyda, Elena Belonovskaya, Siarhei Kirko, Dorota Żyżelewicz, Agnieszka Zakrzeska, Aliaksei Bakunovich, Viktor Rusin, Valentina Moroz

**Affiliations:** 1 Division of Biochemical Pharmacology, Institute of Biochemistry of Biologically Active Compounds, National Academy of Sciences, BLK, 50, 230030 Grodno, Belarus; vu.buko@tut.by (V.B.); ms.belonovskaya@yandex.ru (E.B.); skirko2002@yahoo.com (S.K.); bakunovich.aliaksei@ibiochemistry.by (A.B.); valmor655@gmail.com (V.M.); 2 Department of Biotechnology, University of Medical Sciences, Krakowska 9, 15-875 Bialystok, Poland; a.zakrzeska@wsmed.pl; 3 Department of Biochemistry, Yanka Kupala State University of Grodno, Ozheshko 22, 230023 Grodno, Belarus; zavodnik_ib@grsu.by; 4 Institute of Food Technology and Analysis, Lodz University of Technology, Stefanowskiego 2/22, 90-537 Lodz, Poland; grazyna.budryn@p.lodz.pl (G.B.); dorota.zyzelewicz@p.lodz.pl (D.Ż.); 5 Institute of Molecular and Industrial Biotechnology, Lodz University of Technology, Stefanowskiego 2/22, 90-537 Lodz, Poland; 6 The 2nd Department of Surgical Diseases, Grodno State Medical University, Gorkogo 80, 230009 Grodno, Belarus; rw_2006@mail.ru

**Keywords:** in vivo study, alcoholic steatohepatitis, oxidative stress, inflammatory response, chlorogenic acid

## Abstract

The aim of this study was to evaluate the therapeutic effects of chlorogenic acid (CGA) in rats with advanced alcoholic steatohepatitis. The rats were fed on a high-fat diet and gavaged with ethanol (4 g/kg) for 8 weeks. The livers of ethanol-treated rats showed steatosis; necrosis and mononuclear infiltration; and significant upregulation of the mRNA expression of the prooxidant (Cyp2e1, iNos), lipogenic (Srebp1, Acc), proinflammatory (Tlr4, Nf-κb, TnfA, Il-1B, and Il-6), and profibrogenic (TgfB, Col1, VegfA) genes. Simultaneously, a downregulation of level of *Sod* and *Nrf2* was observed, which was accompanied by increased serum transaminase, TnfA, and serum and liver triglycerides levels. CGA administration (40 and 80 mg/kg, 8 weeks) to ethanol-fed group reduced the liver expression levels of *Cyp2e1* and *iNos*, whereas it markedly enhanced the expression of *Sod*, *Nrf2*, and *Ho-1*. CGA at both doses downregulated the expressions of lipogenic, proinflammatory, and profibrogenic genes, while the expression of *Tlr4* was lowered only after the higher dose of CGA. The higher dose of CGA efficiently prevented the progression of alcohol-induced steatosis and reduced inflammation through regulation of the expression of genes encoding the proteins involved in the Tlr4/Nf-κB signaling pathway and fibrosis. The study revealed hepatoprotective and anti-inflammatory effects of CGA through the regulation of expression of genes encoding Cyp2e1/Nrf2 involved in oxidative stress modulation. These results demonstrate CGA as a therapeutic candidate for the prevention and treatment of alcoholic steatohepatitis.

## 1. Introduction

Alcoholic liver disease (ALD) is a major component of morbidity and mortality in the world. Excessive alcohol intake is the leading cause of ALD, which can progress from fatty liver (steatosis) to steatosis combined with inflammation (steatohepatitis) and further to cirrhosis and end-stage hepatocellular carcinoma. Alcoholic steatosis is a reversible pathology, and its development can be limited by abstinence [1]. In alcoholic steatohepatitis, which is characterized by infiltration of liver tissue with inflammatory cells and elevated hepatic necrosis, the treatment requires the use of drugs with pronounced anti-inflammatory and anti-fibrotic properties.

Over recent years, increasing attention has been paid to phytocompounds as promising therapeutic agents in the treatment of ALD. Phytochemicals can act on various targets for the pathogenetic chain of ALD, with fewer side effects. Several plant-derived substances, e.g., silymarin [2] and curcumin [3], have been previously shown to be quite effective in the treatment of ALD. Some other herbal substances, such as resveratrol [4], genistein, puerarin, and betulin [5], are also promising medicines for the correction of alcohol-dependent liver pathology.

From the large number of experimentally studied plant preparations used for treatment of liver diseases, chlorogenic acid is one of the most promising. Being a major phenolic component in many plants, especially coffee beans, chlorogenic acid (CGA, 5-O-caffeoylquinic acid) is a highly abundant phytochemical present in the human diet. Coffee and plant extracts containing CGA decrease fatty acid synthesis, hepatic inflammation, and the activation of stellate cells [6,7]. Recently, we demonstrated alleviation of thioacetamide-induced liver fibrosis in rats by an extract of CGA-rich green coffee beans, whereas the extract from roasted coffee beans (with a greatly reduced content of CGA) showed a significantly lower antifibrotic effect [8]. CGA is considered as a powerful antioxidant and free radical scavenger, which exhibits a wide spectra of biological activities, including anti-inflammatory, hypolipidemic, and antifibrotic effects [8]. The hepatoprotective effect of CGA was demonstrated in mice fed on a high-fat diet [9] and in a rat model of nonalcoholic steatohepatitis (NASH) [10]. A recently published article demonstrated that, in mice treated with ethanol and CGA (orally, 3 g/kg for 7 days), inhibition of liver steatosis, inflammation, and fibrosis were observed [11]. However, this study did not mention any signs of inflammation in the liver; neither inflammatory infiltration nor necrosis were observed, whereas the mRNA expression of *TnfA* was increased only 1.2-fold in the ethanol-treated group. Moreover, there was also no evidence of liver fibrosis in this group, and the expression of the mRNA encoding markers of liver fibrosis, such as α-Sma and Tnfβ, was changed without statistical significance. Therefore, it should be taken into consideration that this study demonstrated a protective effect of CGA in alcoholic steatosis. The literature data also confirm the absence of inflammation and fibrosis of the liver in subacute alcohol intoxication (intragastric, 2.5 g/kg, 15 days) [12]. Thus, the effect of CGA on advanced alcoholic steatohepatitis still remains unclear.

The antioxidant properties and potential beneficial health effects of CGA, as well as an exact mode of action, metabolism, and possible toxicity and interaction with cellular components are problems of considerable interest. Therefore, the aim of this study was to evaluate the effects of CGA on advanced alcoholic steatohepatitis with the focus on alcohol-specific pathobiology: hepatic lipid accumulation, inflammation, liver fibrosis, and oxidative stress. In order to characterize the mechanism of action of CGA during in vivo studies with albino Wistar rats, chlorogenic acid ability to modulate a wide range of the gene expression encoding proteins involved in ALD pathogenesis was examined.

## 2. Materials and Methods

### 2.1. Chemicals

Chlorogenic acid of 95% purity was purchased from Sigma-Aldrich Co. (St. Louis, MO, USA). All other chemicals and materials were commercially available and of high grade, and they are described below.

### 2.2. Animals & Experimental Design

The care, use, and procedures on the animals conformed to institutional guidelines and were in compliance with national and international laws and the guidelines set forth in Directive 2010/63/EU of the European Parliament and of the Council of 22 October 2010 on the protection of animals used for scientific purposes. The Local Ethics Committee for animal experiments in Grodno and the Ethic Committee of the National Academy of Sciences, Belarus, reviewed and approved the study protocol No 07/18 of 19 April 2018.

Different experimental models of ALD have been used to evaluate the pharmacological activity of the tested hepatoprotective compounds because no models are available that closely mimic human pathology [13]. Bearing in mind that the advanced stages of ALD develop only in a subset of long-term heavy drinkers [14], in this study with rats we applied chronic ethanol intoxication combined with feeding a high-fat diet (HFD) to induce advanced liver injury, which directly mimics human lifestyle and features related liver damage. This model induced steatosis, liver injury, and hepatic neutrophil infiltration, which are synergistically provoked by moderate obesity and alcohol in experimental animals [15,16].

Wistar rats are widely used for the modeling of alcoholic steatohepatitis as they are susceptible to ethanol-induced hepatic steatosis and respond to fibrotic stimuli with the development of a fibrotic reaction [17]. Therefore, in the study, male albino Wistar rats with initial body weight ranging from 200 to 220 g were used. Rats from all the groups (each group consisted of 12 animals) were fed on the HFD ad libitum for 8 weeks. The animals had free access to food and water and were kept under controlled conditions of 22 °C ± 2 °C temperature and of 55% ± 5% relative humidity with a 12-h light/darkness cycle. The basal diet for the control group contained the following components (in %): casein, 21.0; starch, 41.5; fat (lard and sunflower oil, 1:1 wt/wt), 26.0; cellulose, 6.5; and vitamin and mineral mix, 5.0. In case of ethanol-treated groups, the starch was isocalorically substituted by ethanol [18]. Rats of all groups were adapted to a control high-fat diet for 10 days. The experimental protocol of the high-fat diet is presented in Table 1.

Rats were divided into four groups. For 8 weeks, three groups of the animals fed on the HFD were treated with ethanol via oral gavage (4 g/kg b.w., daily between 9:00 and 10:00 a.m.). Throughout the experiment (8 weeks), two of these groups were administered with CGA via oral gavage at dosages of 40 and 80 mg/kg b.w. per day between 4:00 and 5:00 p.m. (groups ASH + CGA, 40 mg/kg and ASH + CGA, 80 mg/kg), respectively. Animals of all group received isocaloric food. Food intake was monitored daily and animals were weighed twice a week. Based on these data, at the beginning of each subsequent week, the components of the diet were recalculated. All groups of animals received a high fat diet (HFD) and water ad libitum during the experimental period. At the end of the experiment, after 12-h fasting, the animals were sacrificed by aorta dissection and exsanguination under general anesthesia achieved via intraperitoneal injection of a pentobarbital solution (50 mg/kg b.w.). Livers were dissected and a portion of fresh liver pieces was used for leucocyte isolation, whereas the second part was frozen and stored in liquid nitrogen for subsequent analysis. The left lobe of the liver (approximately 1 cm^3^ in size) was fixed for histological investigations. Blood was collected after sacrificing and centrifuged for serum preparation.

According to the literature data, the concentrations of CGA used for the correction of liver pathology (steatohepatitis and toxic lesions) ranged from 10–40 mg/kg b.w. [11] to 200 mg/kg b.w. [19]. Focusing on the work, an intermediate dose has been chosen, because doses of 37.5, 75, and 150 mg/kg b.w. of CGA were used to correct cholestatic liver damage [20]. The period of CGA administration was chosen in order to obtain the similarity to the real clinical situation. Herbal preparations, for example, silymarin, are administered to patients with liver pathology for a long time, ranging from 4 weeks to 41 months [21]. Choosing a period of 8 weeks, we focused on the effect of CHA on metabolic syndrome (including fatty liver) in Wistar rats [22].

### 2.3. Biochemical Analysis

The activities of the marker enzymes present in serum (alanine aminotransferase (ALT), aspartate aminotransferase (AST), and alkaline phosphatase), as well as the content of serum and liver triglycerides, were measured according to the manufacturer’s instructions using standard commercial kits from Lachema (PLIVA-Lachema Diagnostika, Brno, Czech Republic). Serum concentrations of rat tumor necrosis factor alpha (Tnfα) were analyzed using commercial ELISA test kits (R&D Systems GmbH, Wiesbaden, Germany). The end-products of lipid peroxidation in liver tissue were measured as thiobarbituric acid–reacting substances (TBARS) [23] and calculated using an extinction coefficient of (1.56 × 10^5^ M^−1^ cm^−1^). Determination of triglycerides in liver tissue was carried out in accordance with the generally accepted protocol [24], guided at the final stage by the instructions of the Kit manufacturer (Erba LaChema, Brno, Czech Republic). Briefly, liver pieces were rapidly frozen in liquid nitrogen. Lipids were saponified using ethanolic KOH (2 parts EtOH: 1 part 30% KOH). After neutralization and centrifugation, a commercial triglyceride Kit was used to measure triglyceride level in the supernatant.

### 2.4. Isolation of Liver Leukocytes

Liver leukocytes were isolated from the liver, minced through mesh and then separated from hepatocytes and debris by Ficoll-Isopaque density (1.090) gradient centrifugation according to Arai et al. [25]. The cells were collected from the interface and the total population of leukocytes was counted in a microscope using Goryaev’s chamber.

### 2.5. Histological Evaluation

Liver samples were randomly selected, fixed in Bouin’s solution, and embedded in paraffin wax. Histological sections were prepared and stained with hematoxylin and eo-sin. Other sections were stained with Sirius Red dye to assess for histological features of fibrosis. Additional liver samples were fixed in Becker solution for histochemical lipid determination, and cryostat sections were fixed and stained with Sudan Black. Tissue sections were imaged with an Olympus CX-41 light microscope using a 40 objective, and the digital images were captured with an Olympus C-5060 camera (Olympus, Tokyo, Japan). The morphometric quantitative evaluation of the sudanophylic area on liver slides characterizing neutral lipids accumulation in the liver was performed with the Image J morphometric analysis software (NIH, Bethesda, MD, USA). To analyze inflammatory infiltration, inflammatory foci consisting of three and more inflammatory cells in ten random fields of view were registered.

### 2.6. Gene Expression Analysis

Hepatic mRNA expression level was determined in liver tissue using quantitative RT-PCR for a set of genes associated with lipogenesis, inflammation, and fibrotic activity, as well as genes mediating cellular antioxidant defenses and oxidant. Total RNA was extracted from liver tissue samples (30 mg) using RNA Extracol reagent and GeneMatrix Universal RNA Purification Kit (Eurex Ltd., Gdansk, Poland) according to the manufacturer’s procedure. RNA samples were purified with Amplification Grade DNase I (Sigma-Aldrich, St. Louis, MO, USA) and reverse transcribed with an NG dART RT Kit (Eurex Ltd., Gdansk, Poland). Real time RT-PCR was carried out using SG qPCR Master Mix (Eurex Ltd., Gdansk, Poland) on a BioRad CFX96 qPCR System (Bio-Rad, Hercules, CA, USA). Complementary DNA representing 6 ng of total RNA per sample was subjected to 25 to 40 cycles of PCR amplification. Samples were first incubated at 95 °C for 40 s, then at 55 °C for 30 s, and, finally, at 72 °C for 30 s. To exclude non-specific products and primer-dimers, after the cycling protocol, a melting curve analysis was performed by maintaining the temperature at 52 °C for 2 s, followed by a gradual temperature increase to 95 °C. The threshold cycle (Ct) values for that gene did not change in independently performed experiments. The level of target gene expression was calculated as 2^−ΔΔCt^, where ΔΔCt = [Ct(target) − Ct(β-actin)]sample − [Ct(target) − Ct(β-actin)] calibrator. The following primer sequences were used to determine the genes’ expression: sterol regulatory element-binding protein 1, *Srebp1* (FP) 5′-TTTTCGTTAACGTGGGTCTCCT-3′, and (RP) 5′-TGGATGGGCAGTTTGTCTGT-3′; acetyl CoA carboxylase, *Acc* (FP) 5′-GTTGGACAACGCCTTCACAC-3′, and (RP) 5′-GCGCATGGAATGGCAGTAAG-3′; AMP-activated protein kinase A, *AmpkA* (FP) 5′-AAGATCGGACACTACGTGCT-3′, and (RP) 5′-TGGAGGCGAGGTAGAACTCA-3′; catalase, *Cat* (FP) 5′-TTTTCACCGACGAGATGGCA-3′, and (RP) 5′-AAGGTGTGTGAGCCATAGCC-3′; nuclear factor kappa B, *Nf-κB* (FP) 5′-CATGGATCCCTGCACACCTT-3′, and (RP) 5′-CTCAGCATGGAGAGTTGGCA-3′; toll-like receptor 4, *Tlr4* (FP) 5′-CGCTTTCAGCTTTGCCTTCA-3′, and (RP) 5′-CTCCAGAAGATGTGCCTCCC-3′; sirtuin 1, *Sirt1* (FP) 5′-GGCAGACAATTTAATGGGGTGA-3′, and (RP) 5′-GAGATCCGGGAAGTCCACAG-3′; tumor necrosis factor α, *TnfA* (FP) 5′-TACTGAGTGTGAGGGTCTGG-3′, and (RP) 5′-ATGCTGAGGTTGGACGGATA-3′; interleukin 1, *Il-1* (FP) 5′-GACTTCACCATGGAACCCGT-3′, and RP: 5′-GGAGACTGCCCATTCTCGAC-3′; interleukin 6, *Il-6* (FP) 5′-AGCGATGATGCACTGTCAGA-3′, and (RP) 5′-TAGCACACTAGGTTTGCCGA-3′; heme oxygenase 1, *Ho-1* (FP) 5′-TTAAGCTGGTGATGGCCTCC-3′, and (RP) 5′-GTGGGGCATAGACTGGGTTC-3′; cyclooxygenase-2, *Cox-2* (FP) 5′-TGAGTACCGCAAACGCTTCT-3′, and RP: 5′-ACACAGGAATCTTCACAAATGGA-3′; Cu-Zn superoxide dismutase, *SOD* (FP) 5′-TAACTGAAGGCGAGCATGGG-3′, and (RP) 5′-CCTCTCTTCATCCGCTGGAC-3′; cytochrome P450 2E1, *Cyp2e1* (FP) 5′-TTCACCAAGTTGGCAAAGCG-3′, and (RP) 5′-AGGCTGGCCTTTGGTCTTTT-3′; glutathione-disulfide reductase, *Gsr* (FP) 5′-TACTGCACTTCCCGGTAGGA-3′, and (RP) 5′-TGGATGCCAACCACCTTCTC-3′; glutathione peroxidase, *Gpx* (FP) 5′-CAGTCCACCGTGTATGCCTT-3′, and (RP) 5′-GTAAAGAGCGGGTGAGCCTT-3′; nuclear factor erythroid 2-related factor 2, *Nrf2* (FP) 5′-CATTTGTAGATGACCATGAGTCGC-3′, and (RP) 5′-CGGTGGGTCTCCGTAAATGG-3′; transforming growth factor β, *TgfB* (FP) 5′-ATGACAGCCCAACCAAGGAA-3′, and (RP) 5′-TCGAGTGATGGAATGTGCGT-3′; collagen, type I, alpha 1, *Col1* (FP) 5′-AAGGCTCCCCTGGAAGAGAT-3′, and (RP) 5′-CAGGATCGGAACCTTCGCTT-3′; vascular endothelial growth factor A, *VegfA* (FP) 5′-ACTCATCAGCCAGGGAGTCT-3′, and (RP) 5′-GAGCCCAGAAGTTGGACGAA-3′; inducible nitric oxide synthase, *iNos* (FP) 5′-AGAGGAGGACGCTGGTGTAT-3′, and (RP) 5′-AACAGGACAAGAGGCAGAGC-3′; *β*-actin, *B-Act* (FP) 5′-ATCATTGCTCCTCCTGAGCG-3′, and (RP) 5′-GAAAGGGTGTAAAACGCAGCTC-3′.

### 2.7. Statistical Analysis

Determination of average values and one-way ANOVA analysis, followed by the Dunnett’s test expressed as means ± standard deviation (M ± SD), were performed using the GraphPad Prism 6.0 software (GraphPad Software, Inc., La Jolla, CA, USA), where *p* value of ≤0.05 was considered significant.

## 3. Results

### 3.1. Effect of CGA on Liver Morphology and Histopathologic Changes

The liver/body mass ratio was markedly elevated in ethanol-treated rats, and it decreased after the administration of both doses of CGA (Table 2).

**Figure 1:**
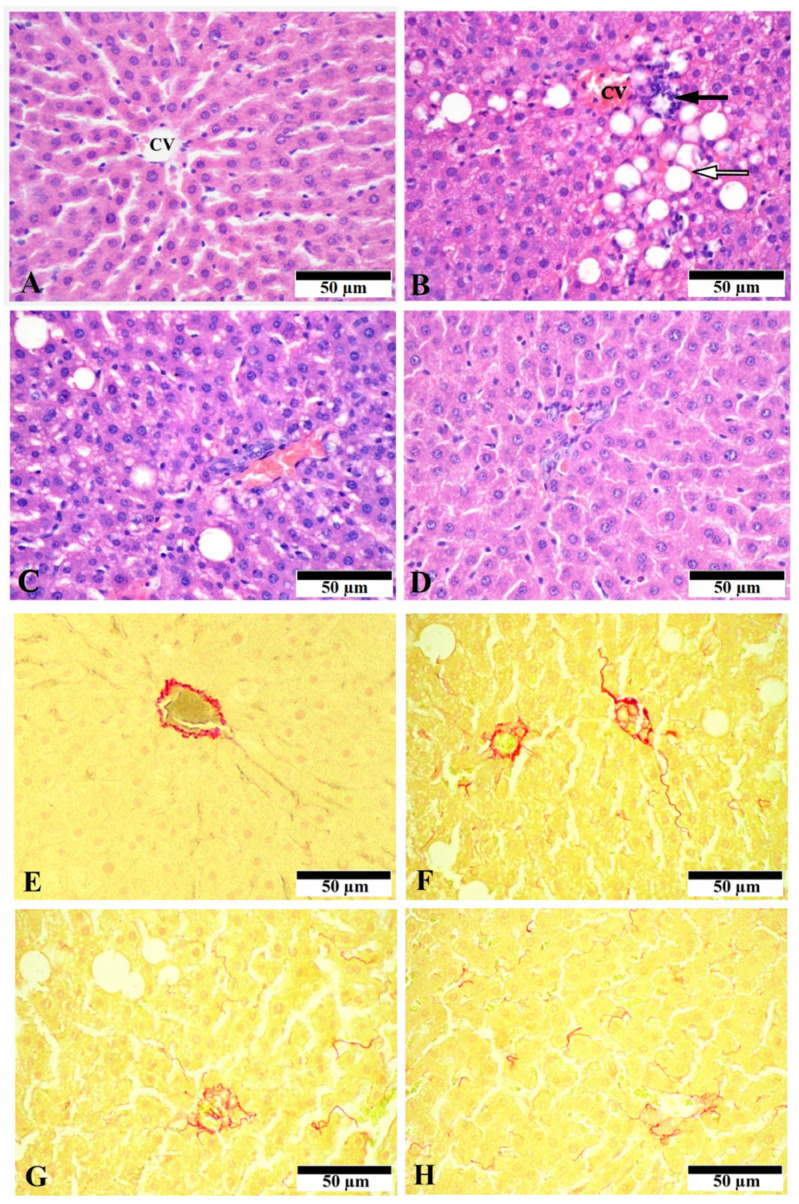
Representative photographs of liver slides. (**A**–**D**): Staining with haemotoxylin and eosin, original magnification × 400. (**A**)—Control group showed the normal liver architecture, cell structure, and central vein (CV) with no abnormalities; (**B**)—Alcohol-treated group (ASH). Macrovesicular fatty dystrophy (white arrow) and inflammatory foci in the ASH group (black arrow); (**C**)—ASH + CGA, 40 mg/kg; (**D**)—ASH + CGA, 80 mg/kg. In the groups treated with CGA, mainly microvesicular steatosis and scattered lymphoid infiltration were seen. (**E**–**H**): Staining with Sirius Red, original magnification × 400. (**E**)—Control group with no abnormalities. Connective tissue was observed only in the walls of major blood vessels and hepatic capsule. (**F**)—Alcohol-treated group (ASH). Moderate collagen deposition around central veins and portal areas. (**G**)—ASH + CGA, 40 mg/kg; (**H**)—ASH + CGA, 80 mg/kg. The decrease of collagen deposition.

The histological evaluation of control livers showed the normal hepatic structure without pathological signs (Figure 1A). The liver slides from the ethanol-treated group demonstrated apparent steatosis, which was mainly macrovesicular and, to a lesser extent, microvesicular. Necroinflammation in the liver ranged from moderate mononuclear infiltration, which was most prominent around the central veins, to manifold multifocal infiltrates with individual cell necrosis in the liver parenchyma and scattered parenchymal cells with ballooning degeneration (Figure 1B). A significant improvement of the histological pictures in rats treated with CGA, especially with its higher dose (80 mg/kg), was observed. Livers form these groups were mainly characterized by microvesicular steatosis and scattered lymphoid infiltration (Figure 1C,D).

**Figure 2:**
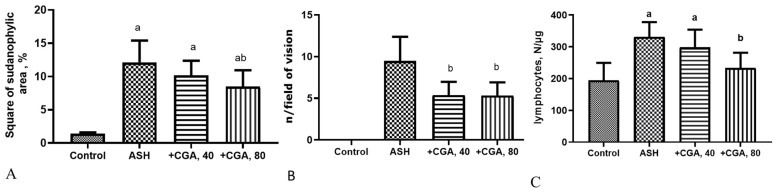
Results of morphometric evaluation of liver slides. (**A**)—The relative square of the sudanophylic area reflects accumulation of neutral lipids into hepatocytes; (**B**)—The number of inflammatory foci per the field of vision. (**C**)—The inflammatory cells (leucocytes) count in the liver (number of cells/mg tissue); M ± SD; a—*p* < 0.05 compared to the control group; b—*p* < 0.05 compared to the ethanol-administered group.

Sirius Red staining showed slight deposits of connective tissue in the region of the capsule, portal tracts, and the wall of the central veins of the hepatic lobules in the control animals (Figure 1E), which corresponds to the normal state. The liver slides from the ethanol-treated group demonstrated moderate accumulation of extracellular matrix around central veins and portal areas as well as signs of pericellular fibrosis, whereas the treatment with CGA showed decreased collagen deposition compared with the ASH group (Figure 1F–H). The staining with Sudan Black detected the massive presence of black-stained lipid droplets in the alcohol-administered group, where the relative area of sudanophylic staining 8-fold exceeded the control values (Figure 2A). The number of inflammatory foci per the field of vision in this group was of 9.5 ± 2.78 (Figure 2B), indicating a high degree of inflammatory response. The number of inflammatory cells (leukocytes) per mg of liver tissue dramatically increased in the ethanol-treated group (more than 5.5-fold) as compared to control animals (Figure 2C).

The treatment of the ethanol-fed rats with CGA, especially with its higher dose (80 mg/kg), dramatically reduced liver steatosis, diminishing the size and the amount of lipid droplets in hepatocytes. The morphometric measurement of the sudanophylic area of liver slides, which reflects neutral lipids accumulated in the liver cells, showed a statistically significant decrease of this parameter only in rats treated with the higher dose of CGA (80 mg/kg) (Figure 2A). Both doses of CGA equally reduced the number of inflammatory foci in the liver (Figure 2B). However, when the highest dose of CGA was administered, the number of inflammatory cells in the liver was significantly lower, approaching the control level (Figure 2C). Collectively, the morphological data demonstrate that the CGA treatment attenuated steatosis and inflammation in the rat alcoholic liver.

### 3.2. Effect of CGA on Serum and Liver Biochemical Parameters

For assessment of the effect of CGA on hepatocellular damage, the activity of serum marker enzymes was measured. All the activities associated with liver injury, such as AST, ALT, and alkaline phosphatase were significantly elevated in alcohol-fed rats (Table 2). Both doses of CGA (40 and 80 mg/kg) decreased these enzymes activities to a similar extent. The amount of serum and liver triglycerides was elevated in rats intoxicated with alcohol and equally diminished in rats treated with both doses of CGA. The serum level of Tnfα and Il-6, proinflammatory cytokines, was significantly elevated in the ethanol-fed group (each nearly 2-fold), whereas only the highest dose of CGA (80 mg/kg) decreased these parameters with statistical significance. The TBARS content was increased (2.6 fold) in the liver of animals from the ASH group (Table 2). Both CGA doses lowered this parameter similarly, bringing it closer to the control level.

### 3.3. Effects of CGA on Hepatic Gene Expression of Nrf2, Prooxidant and Antioxidant Enzymes

**Figure 3:**
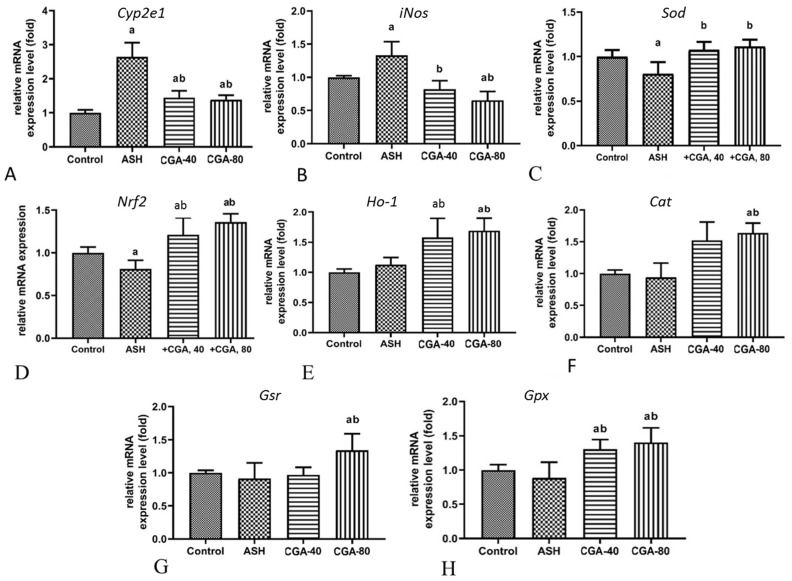
Effect of two chlorogenic acid doses (40 and 80 mg/kg) on the expression of prooxidant and antioxidant genes in the liver of rats with alcoholic steatohepatitis (ASH) induced by chronic alcohol intoxication. (**A**)—Cyp2e1; (**B**)—iNos; (**C**)—Sod; (**D**)—Nrf2; (**E**)—Ho-1; (**F**)—Cat; (**G**)—Gsr; (**H**)—Gpx. M ± SD. a—*p* < 0.05 compared to the control group; b—*p* < 0.05 compared to the ethanol-administered group.

Raised levels of the mRNA expression of Cyp2e1, inducible nitric oxide synthase (iNos), were found in the liver of rats with chronic alcohol intoxication (Figure 3A–C).The ethanol administration significantly downregulated the relative mRNA gene expression level of Nrf2, the key transcription factor regulating antioxidant enzymes, Cu-Zn superoxide dismutase (Sod), and did not affect other downstream target genes such as heme oxygenase 1 (Ho-1), catalase (Cat), glutathione reductase (Gsr), and glutathione peroxidase (Gpx) (Figure 3D–H). Comparatively, the treatment of ethanol-administered rats with both doses of CGA (40 and 80 mg/kg) prevented response to alcohol intoxication, reducing the mRNA expression levels of Cyp2e1 and iNos, and increasing the level of Sod. Moreover, both doses of CGA upregulated the expression of genes encoding Nrf-2, Ho-1, Cat, and Gpx, whereas the expression level of Gsr was increased only in the group treated with the highest dose of CGA (80 mg/kg).

### 3.4. Effects of CGA on Inflammation-Related Gene Expression

**Figure 4:**
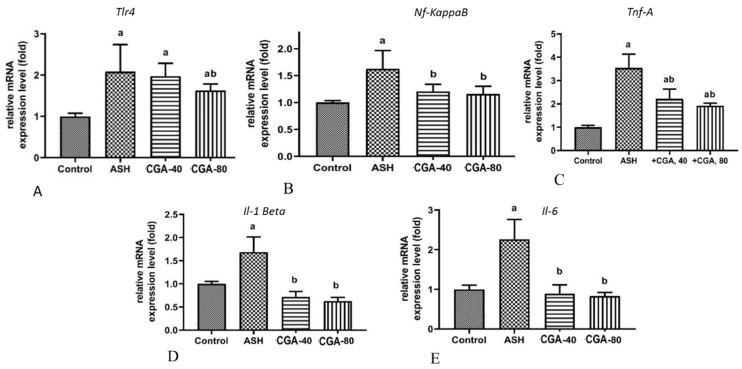
Effect of two CGA doses (40 and 80 mg/kg) on the expression of inflammation-related genes in the liver of rats with alcoholic steatohepatitis (ASH) induced by chronic alcohol intoxication. (**A**)—Tlr4; (**B**)—Nf-kB; (**C**)—Tnfα; (**D**)—Il-1β; (**E**)—Il-6. M ± SD. a—*p* < 0.05 compared to the control group; b—*p* < 0.05 compared to the ethanol-administered group.

The tool-like receptor 4 (TLR4), a key trigger of the inflammatory process, and the nuclear factor-κB (NF-κB), an oxidative stress-mediated transcriptional factor, regulate an expression of a large number of proinflammatory genes and mediate inflammatory response due to the production of proinflammatory cytokines [26]. Compared to healthy rats, chronic alcohol intoxication significantly upregulated the mRNA of genes expressing Tlr4 and Nf-κB (Figure 4A,B) and, subsequently, the expression of the proinflammatory cytokines, TnfA, Il-1, and Il-6 (Figure 4C–F). The effect of ethanol on TnfA was especially pronounced (more than 3.5-fold increase). Both doses of CGA randomly decreased the expression of all the above proinflammatory genes on the mRNA level: Nf-kB, TnfA, Il-1, and Il-6, with statistical significance, whereas the mRNA expression of the gene encoding Tlr4 was significantly upregulated only with the highest dose of CGA (80 mg/kg).

### 3.5. Effects of CGA on Expression of Genes Related to Lipid Metabolism

**Figure 5:**
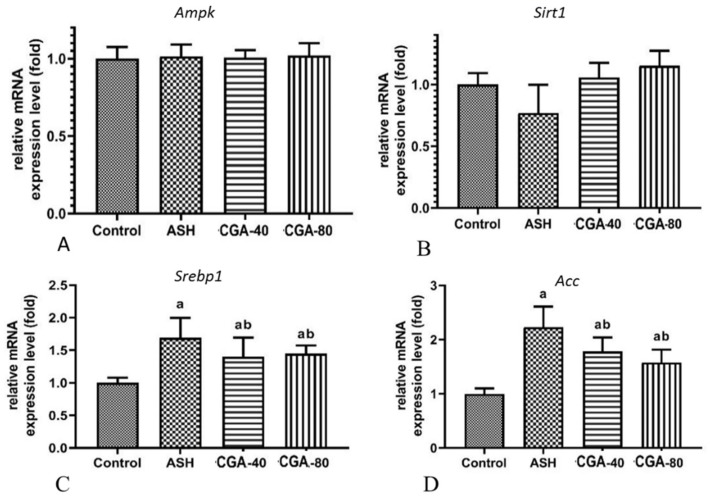
Effect of two CGA doses (40 and 80 mg/kg) on the expression of genes related to lipid metabolism in the liver of rats with alcoholic steatohepatitis (ASH) induced by chronic alcohol intoxication. (**A**)—Ampkα; (**B**)—Sirt1; (**C**)—Srebp1; (**D**)—Acc. M ± SD. a—*p* < 0.05 compared to the control group; b—*p* < 0.05 compared to the ethanol-administered group.

It was suggested that lipogenesis in ALD is stimulated via the AMP-activated protein kinase (AMPK)/sirtuin 1 (SIRT1) signaling pathway, which further regulates the transcriptional activity of sterol element-binding protein 1 (SERBP1) and its target genes, such as lipogenic enzymes [27]. In all of the animal groups studied, we did not find any significant changes in the expression level of mRNA encoding genes of either subunit α of AMPK or SIRT1 genes (Figure 5A,B).

The presented data suggest that during experiment conditions the observed liver steatosis was not followed by the changes on the expression level of genes encoding the AMPK or SIRT1 proteins, which are known as the major regulators of cellular lipid and glucose metabolism. However, the mRNA level of Srebp1 and acetyl-CoA carboxylase (Acc) were significantly elevated in the ethanol-administered group (Figure 5C,D). The ACC protein catalyzes carboxylation of acetyl-CoA to malonyl-CoA and is known as the critical enzyme in the fatty acid biosynthesis pathway. Both doses of CGA (40 and 80 mg/kg) similarly downregulated Srebp1 and Acc expression levels, suggesting that the pronounced ani-lipogenic effect might be mediated on the transcription level.

### 3.6. Effects of CGA on Expression of Genes Related to Liver Fibrosis

**Figure 6:**
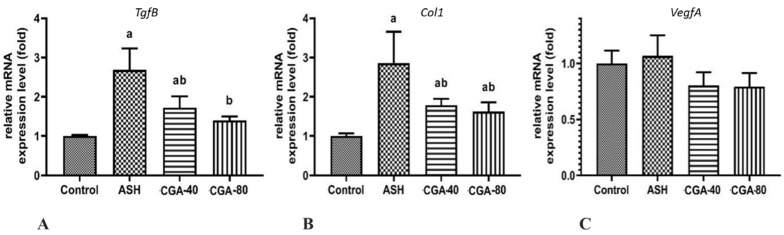
Effect of two CGA doses (40 and 80 mg/kg) on the expression level of genes related to fibrogenesis in the liver of rats with alcoholic steatohepatitis (ASH) induced by chronic alcohol intoxication. (**A**)—TgfB; (**B**)—Col1; (**C**)—VegfA. M ± SD. a—*p* < 0.05 compared to the control group; b—*p* < 0.05 compared to the ethanol-administered group.

The administration of ethanol significantly elevated (more than 2.7-fold) the mRNA level of gene encoding transforming growth factor beta (TGFβ) (Figure 6A), the key profibrogenic factor activating hepatic stellate cells to myofibroblasts and driving liver fibrosis [28]. Type 1 collagen (COL1) is the main component of the extracellular matrix in alcoholic liver fibrosis [29], and its gene expression on transcription level was also markedly increased (about 2.9-fold) in the liver of rats with chronic alcohol intoxication (Figure 6B).

Angiogenesis preceding progression of liver fibrosis is regulated by angiogenic cytokines, among which vascular endothelial growth factor alpha (VEGFα) plays the most prominent role [30]. Chronic alcohol intoxication did not significantly affect the VegfA in the rat liver on the transcription level (Figure 6C). In this case, the expression of mRNA encoding all the above genes was similarly downregulated by both doses of CGA (40 and 80 mg/kg) (Figure 6A–C), suggesting pronounced antifibrogenic activity of the studied compound, even at the lower dose of 40 mg/kg.

## 4. Discussion

In the current study, rats fed on the HFD were exposed to alcohol intoxication for an extended period of time in order to induce chronic liver damage resembling that of chronic ALD in humans. Our model reproduced the main pathogenetic features in alcoholic steatohepatitis, as described previously [18,23]. After 8 weeks of the intragastric ethanol administration, accompanied by HFD feeding, the rats showed advanced steatohepatitis, as evidenced by the increased liver/body weight, and dramatically elevated enzymes ALT, AST, and alkaline phosphatase activities, as well as hepatic steatosis and inflammation. Therefore, the effects of CGA on these pathological changes were examined. We found that the CGA administration adequately protected rats against alcoholic liver injury, reducing serum ALT and alkaline phosphatase activities, as well as decreasing serum and hepatic triglyceride levels (Table 2) and the formation of lipid droplets in hepatocytes (Figure 2). The effect of CGA on the liver inflammation was especially pronounced by the decreased number of hepatic inflammatory foci and leukocytes.

Chronic alcohol intoxication was associated with an increase in the production of reactive oxygen species (ROS) in the liver and caused disturbances in the hepatic antioxidant defense. This, in turn, led to oxidative stress playing the critical role in the pathogenesis of alcoholic liver disease [31]. Most authors generally explain the hepatoprotective effects of CGA by its antioxidant properties, because chlorogenic acid is a powerful scavenger of different kinds of ROS [32]. In particular, the protective effects of CGA in acetaminophen toxicity [33], CCl_4_-induced liver fibrosis [34], and ischemia/reperfusion injury [35] were mechanistically linked to inhibition of oxidative stress. After the short-term alcohol exposure (3 g/kg for 1 week), the treatment with CGA also alleviated liver steatosis by reducing the accumulation of intracellular ROS and the lipid peroxidation product, 4-hydroxynonenal, and inhibiting oxidative stress [11]. In our study, the treatment of alcoholic steatohepatitis with both doses of CGA significantly decreased the level of TBARS, which are the end-products of lipid peroxidation in the liver (Table 2).

The main source of ROS in the alcohol-damaged liver is the ethanol-inducible isoform of cytochrome P-450, CYP2E1 [36]. Moreover, CYP2E1 participates in ethanol metabolism, producing its toxic and highly reactive metabolites, acetaldehyde, and 1-hydroxyethyl radical, which in turn can modify liver DNA and promote ethanol hepatotoxicity [37]. The literature data suggest that the Cyp2e1 mRNA expression in the liver of experimental animals chronically treated with ethanol alone [38] and ethanol combined with HFD [39] was up-regulated up to 4–5-fold. In our study, chronic alcohol intoxication for 8 weeks induced pronounced oxidative stress, elevating not only the expression of Cyp2e1 on the mRNA level (2.5 fold), but also *iNos* encoding the protein that produces reactive nitrogen species involved in the pathogenesis of alcoholic liver injury [40]. In addition, the long-term administration of ethanol decreased expressions of *Nrf2*, the main regulator of antioxidant enzymes, and *Sod*, the major antioxidant enzyme that inactivates the highly reactive superoxide anion. It should be noted that expression at the mRNA level of most of the other genes responsible for protection against oxidative stress, such as Nrf2, Ho-1, Gpx, Gsr, and Cat, were not changed in the liver during chronic alcohol intoxication. In vitro, CGA was shown to inhibit the formation of the 1-hydroxyethyl radical in liver microsomes [41], which is generated in a CYP2E1-dependent manner [42]. CGA ameliorated oxidative stress and reduced the expression of CYP2E1 in mice with cisplatin-induced kidney injury [43]. In our study, we found that both doses of CGA similarly down-regulated (ca. 2-fold) the *Cyp2e1* expression level in the liver, leading to the alleviation of alcohol-induced steatohepatitis. Based on these results and prior reports, we can consider CGA as a potent inhibitor of CYP2E1. As shown previously, the administration of CYP2E1 inhibitors efficiently suppressed ethanol-mediated lipid peroxidation and boosted antioxidant capacity in the liver [44]. In the current study, we demonstrated that both doses of CGA efficiently decreased the liver TBARS content and upregulated the expression of mRNA encoding the antioxidant enzymes, SOD, CAT, and HO-1, whereas the expression of the two glutathione-related enzymes, *Gsr* and *Gpx*, was elevated only in animals co-treated with the higher dose of CGA (80 mg/kg).

Most detoxification enzymes and antioxidant proteins are controlled by nuclear factor erythroid-2-related factor 2 (Nrf2). The Nrf2, stimulated in oxidative stress, translocates from cytosol to the nucleus, where it binds to the antioxidant response elements and thus upregulates expression of a number of antioxidant and detoxifying genes encoding proteins such as SOD, CAT, HO-1, GPX, and GSR [45,46]. Among these genes upregulated by Nrf2, HO-1, a very powerful anti-inflammatory enzyme, plays a prominent protective role in oxidative stress and inflammation. Many authors emphasize the important role of the Nrf2/HO-1 pathway in hepatoprotection against ethanol-induced liver damage. Different herbal substances having antioxidant activity, such as the flavonoids quercetin [47], curcumin, and baicalin [48], and the pentacyclic-triterpene compound taraxasterol [39], ameliorate ethanol-induced liver injury via the Nrf2/HO-1 pathway. In this regard, the expression of HO-1 increases as a result of Nrf2 factor activation. Therefore, many plant-derived substances protect the liver against alcohol-induced oxidative stress and injury by activation of the Nrf2 transcription factor with subsequent upregulation of HO-1 and other downstream cytoprotective target genes. Similarly to other herbal substances with hepatoprotective properties, CGA enhanced Nrf2 activation and HO-1 expression in rats with CCl_4_-induced liver injury [49]. In the present study, we observed that CGA increased the mRNA expression of Nrf2 and its target genes, among which there are HO-1 and several other downstream genes encoding enzymes functionally involved in antioxidant defense such as SOD, CAT, and GSH. These results confirmed that the hepatoprotective mechanism of CGA in liver injury induced by chronic alcohol ingestion are, at least partially, realized through upregulation of the Cyp2e1/Nrf2/target antioxidant defense genes signaling pathways.

The regulation of inflammatory response, a key mediator in the ALD progression from fatty liver to steatohepatitis, is known to be mediated by both the NF-κB and Nrf2 signaling pathways. Alcoholic liver injury is associated with upregulation of inflammatory cytokines, including TNFα, IL-1β, and IL-6. The initiation of the inflammatory cascade is realized via activation of NF-κB [50]. The NF-κB protein, which is activated in oxidative stress, causes an elevation of inflammatory gene expression, such as cytokines, chemokines, and other contributors of inflammatory responses. In rats treated with CGA, the mRNA expression of *Nf-κB* was significantly decreased and accompanied by upregulation of *Nrf2* expression, which can be explained as a defensive response and suggest a cross-talk between the NF-κB and Nrf2 pathways [51]. In turn, the Nrf2 activation reduced the expression of inflammatory genes. The Nrf2/HO-1 signaling pathway downregulates cytokines and other inflammatory mediators, thus playing a major role in anti-inflammatory function [51].

On the other hand, chronic alcohol consumption disturbs gut permeability and promotes bacterial lipopolysaccharides (LPS) translocation from the gut to the liver, a process that plays a crucial role in inflammatory response. LPS promotes progression of ALD from alcoholic steatosis to steatohepatitis, activating macrophages, especially Kupfer cells, to produce ROS and various cytokines and chemokines. The stimulation of Kupfer cells by LPS is mediated by LPS-specific toll-like receptor 4 (TLR4), which is involved in inflammatory response observed in alcoholic liver damage [52]. The LPS-triggered activation of TLR4 leads to an increase in NF-κB expression, resulting in the overproduction of proinflammatory cytokines including TNFα, IL-1, and IL-6 [53]. Based on our results, it can be found that chronic alcohol intoxication enhanced the expression of *Tnfα*, *Il-1*, and *Il-6* on transcription level in the rat liver, as well as the circulating (serum) concentration of TNFα and IL-6. The treatment with CGA, especially with the highest dose, markedly decreased all the above proinflammatory parameters. These effects appear to be linked to upregulation of both Nrf2 and *Ho-1* and downregulation of *Nf-κB* and *Tlr4* expressions, whereas the *Tlr4* expression decreased significantly only in the group treated with CGA (80 mg/kg). It can be suspected that CGA attenuates inflammatory response involving the Nrf2/HO-1 and TLR4/NF-κB signaling pathways in ethanol-administered rats. The above results, together with histological and morphometric data, suggest a pronounced anti-inflammatory effect of CGA in ALD.

Our data demonstrate that chronic alcohol intoxication caused hepatic steatosis, as confirmed by the increased serum marker enzyme activities, serum and hepatic triglyceride concentrations, and the morphological changes characterized by enhanced accumulation of lipids in hepatocytes (lipid droplets and the increase in the sudanophilic area), whereas the administration of CGA at both doses improved these signs of steatosis. Chronic alcohol intoxication stimulated lipogenesis, which is recognized as one of the mechanisms for alcoholic steatosis [54]. The ethanol-induced lipid accumulation into hepatocytes is dependent on the activation of the transcriptional factor, sterol regulatory element-binding protein 1, that controls the biosynthesis of fatty acids, triglycerides, and cholesterol. The ethanol-stimulated activation of *Srebp1* was associated with triglyceride accumulation in the liver and increased expression of genes encoding wide spectra of lipogenic enzymes [55]. In our study, we showed that the expression of *Srebp1*, and its target gene encoding *Acc* catalyzing the first step in lipogenesis, was markedly enhanced in rats with chronic alcohol intoxication. The treatment with both doses of CGA downregulated the *Srebp1* and *Acc* levels in the liver of alcohol-treated rats, which in turn resulted in decreased lipid synthesis and prevention of lipid accumulation as well as reduced liver triglycerides. It can be assumed that CGA reduces the mRNA expression encoding, not only of ACC, but also of other lipogenic enzymes regulated by *Srebp1*, such as fatty acid synthetase, stearoyl-CoA desaturase 1, mitochondrial glycerol-3-phosphate acyltransferase, and ATP citrate lyase [55]; however, this hypothesis needs further study.

The data from several laboratories suggested the role of the AMPK/SIRT1 signaling pathway in regulation of SREBP1 and lipogenic genes in the progression of ethanol-induced steatosis [27,56,57]. However, in our study we did not find any changes in either *AMPKα* or *SIRT1* expression level in any of the CGA-treated groups. It is known that protein phosphorylation by AMP-kinase inactivates the number of metabolic enzymes and other factors involved in lipid metabolism [58]. Because some plan-derived extracts rich in chlorogenic acid have been demonstrated to activate AMPK [58], our results obtained on the transcription level do not exclude involvement of the AMPK signaling. The AMPK kinase is the master regulator of cellular metabolism that regulates the activity of many proteins by their phosphorylation. Thus, further studies need to be performed to check the level of phosphorylated by AMPK target proteins such as pACC or pSREBP1, which, after phosphorylation, should exhibit decreased activity.

Some authors have described that the inhibition of the TLR4/NF-κB signaling pathway, which is related to inflammatory response in chronic liver damage, also helps to protect hepatocytes against steatosis and fibrosis [59,60]. Chronic alcohol intoxication promotes hepatic inflammation by activating Kupffer cells, which play a crucial role in early-stage ALD via the production of inflammatory cytokines and chemokines through TLR4 mediated signaling. The TLR4 signaling also regulates the activation of hepatic stellate cells producing ECM due to inhibition of miR-29 expression [61]. Kupffer cells are centrally involved in regulation of the NF-κB pathway and hepatic inflammatory response. Inactivation of Kupffer cells with gadolinium chloride resulted in a decrease of NF-κB DNA binding activity [62] and the mRNA expression of NF-κB [63], which prevents the development of alcoholic steatosis [64] and confirms that the TLR4/NF-κB signaling pathway can trigger lipid metabolism in ALD. Moreover, TNFα, a proinflammatory cytokine that is controlled by the TLR4/NF-κB pathway, increases fat deposition in the liver, thus upregulates *SREBP1* gene expression [63,64,65,66]. Consequently, we showed that in the liver of rats with chronic alcohol intoxication, CGA attenuated steatosis by decreasing the mRNA expression of the lipogenic genes, *Srebp1* and *Acc*. We hypothesize that CGA acts on lipid metabolism through the TLR4/NF-κB signaling pathway to reduce lipogenesis by downregulation of expression of genes encoding SREBP1 and other related proteins in the liver.

Previously, we have described the antifibrotic effects of extracts from different coffee and cocoa beans where the effect of the extract of green coffee beans containing the highest amount of CGA acted the most effectively [7]. The data from other laboratories showed that CGA reduced liver fibrosis induced by CCl_4_ [67], schistosomiasis [20], or bile duct ligation [62,66]. Some authors proposed that the antifibrotic effect of CGA was connected with inhibition of inflammatory response due to the attenuation of NF-κB signaling and, subsequently, hepatic fibrosis [68]. Similarly, prevention of liver fibrosis with CGA administration in CCl_4_-intoxicated rats is also connected with anti-inflammatory properties of this phenolic compound due to the inhibition of the TLR4/NF-κB signaling pathway [69]. In our study, we showed that chronic ethanol administration in the liver of experimental rats raised, not only the expression of the mRNA levels of proinflammatory cytokines, but also the mRNA expression of the central profibrogenic cytokine TgfB, as well as Col1, the hallmark of fibrosis. Both these parameters were lowered in rats administered with CGA in our model. These data are supported by the results of a histological study with Sudan red staining, where CGA decreased collagen deposition (Figure 1E–H). It could be suspected that the anti-inflammatory effect of CGA is one of the main mechanisms to attenuate liver fibrosis in our system because the mRNA expression of both Tlr4 and NF-κB was also upregulated in the ethanol-administered group, and it decreased in the CGA-treated rats. Additionally, the animal treatment with CGA significantly inhibited the mRNA expression of *VegfA*, a potent inducer of angiogenesis and wound healing. In addition, CGA downregulated the mRNA expression of nitric oxide synthase, which modulates VEGF to promote neovascularization [70]. Angiogenesis contributes to the pathogenesis of liver fibrosis and is closely associated with scar formation preceding the spread of extracellular matrix in the liver. Therefore, the observed anti-angiogenic potential of CGA can contribute to the antifibrotic action of this compound.

The study’s uniqueness exists in the fact that, in rats treated with ethanol, both doses of CGA (40 and 80 mg/kg) had a similar effect on most of the parameters studied. Still, in some experiments, the lower dose of CGA was insignificant to stimulate biological response, i.e., in the expression level of Tlr4 mRNA, the area of sudanophilic staining of liver sections, or the amount of inflammatory cells in the liver. However, the presented study has some limitations that need to be considered, such as the lack of the results on the protein level confirming conclusions based on the results from transcription regulation.

**Figure 7:**
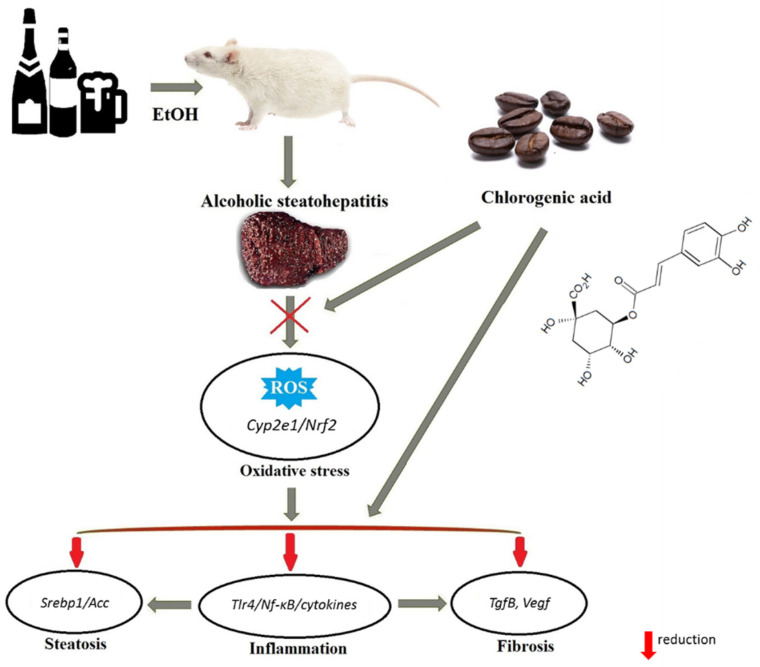
Chlorogenic acid effect on the steatohepatitis in rats with chronic alcohol intoxication—the proposed mechanism of action. CGA possesses protective activity against ROS generation, diminishes the release of inflammatory cytokines, and decreases the fibrosis and steatosis.

In conclusion, our findings demonstrate that long-term treatment with CGA ameliorates steatohepatitis in rats with chronic alcohol intoxication (Figure 7). CGA markedly improved the progression of steatosis, inflammation, and some signs of fibrosis in the liver, induced by ethanol consumption. The beneficial effect of CGA appears to be mostly driven by the improvement in ethanol-induced oxidative stress via regulation of expression of the Cyp2e1/Nrf2/target antioxidant defense. Furthermore, the antioxidant action of CGA inhibited the inflammatory response through the regulation of the expression of genes encoding Tlr4/Nf-κB and the downstream targets: TnfA, Il-1, and Il-6. Taken together, our findings suggest that CGA is a promising multitarget therapeutic candidate for the prevention and treatment of alcoholic steatohepatitis; however, its usage requires further, more detailed, studies.

## Figures and Tables

**Table 1:** Composition of experimental high-fat diet.

	Diet	
	Control Group Diet	EtOH-Treated Groups Diet
Sunflower oil	13	13
Lard	13	13
Cellulose	6.5	12
Corn starch	41.5	35.5
Mineral mix ^1^	3.5	3.5
Vitamin mix ^2^	1.0	1.0
DL-methionine	0.3	0.3
Choline chloride	0.2	0.2

^1^ AIN-93-MX mineral mixture; ^2^ AIN-93-VX vitamin mixture.

**Table 2:** Liver/body mass and serum and liver biochemical parameters in alcohol-fed rats (EtOH) administered with chlorogenic acid (CGA).

	Control	EtOH	EtOH + CGA, 40 mg/kg	EtOH + CGA, 80 mg/kg
Liver/body mass × 100	2.69 ± 0.07	3.15 ± 0.06 ^a^	2.96 ± 0.05 ^ab^	2.88 ± 0.06 ^b^
AST, IU/L	92.2 ± 8.71	135.9 ± 19.63 ^a^	113.8 ± 14.12 ^b^	112.9 ± 17.14 ^b^
ALT, IU/L	50.7 ± 7.23	80.2 ± 20.15 ^a^	59.8 ± 9.32 ^b^	60.1 ± 10.77 ^b^
Alkaline phosphatase, IU/L	102.2 ± 14.2	218.9± 69.4 ^a^	166.0 ± 28.7 ^ab^	144.2 ± 31.1 ^ab^
Serum triglycerides, mmol/L	2.89 ± 0.149	4.12 ± 0.305 ^a^	3.40 ± 0.376 ^ab^	3.24 ± 0.263 ^ab^
Liver triglycerides, mg/g tissue	1.41 ± 0.30	4.83 ± 0.77 ^a^	3.34 ± 0.43 ^ab^	3.04 ± 0.58 ^ab^
Serum TNFα, pg/mL	90.2 ± 2.95	179.8 ± 9.82 ^a^	164.0 ± 8.07 ^a^	144.5 ± 12.44 ^ab^
Serum Il-6, pg/mL	88.9 ± 2.06	181.6 ± 8.61 ^a^	145.2 ± 16.22 ^a^	102.0 ± 5.05 ^ab^
Liver TBARS, nmol/g tissue	39.9 ± 6.06	104.6 ± 15.02 ^a^	59.3 ± 6.53 ^ab^	52.3 ± 4.87 ^ab^

Note: a—*p* < 0.05 as compared to the control group; b—*p* < 0.05 as compared to the alcohol-treated group (ASH).

## Data Availability

Not applicable.
